# Prognostic value of kappa free light chain index in patients with primary progressive multiple sclerosis

**DOI:** 10.3389/fimmu.2025.1658182

**Published:** 2025-11-07

**Authors:** Martin Schmidauer, Klaus Berek, Michael Auer, Gabriel Bsteh, Paola Cavalla, Franziska Di Pauli, Massimiliano Di Filippo, Florian Deisenhammer, Andreja Emeršič, Fabian Föttinger, Lorenzo Gaetani, Michaela Hassler, Nik Krajnc, Dejan Milosavljevic, Markus Ponleitner, Thor Petersen, Stefan Presslauer, Igal Rosenstein, Uroš Rot, Caroline Winther Tørring, Domizia Vecchio, Marco Vercellino, Tobias Zrzavy, Anne Zinganell, Janette Walde, Harald Hegen

**Affiliations:** 1 Department of Neurology, Medical University of Innsbruck, Innsbruck, Austria; 2 Department of Neurology, Medical University of Vienna, Vienna, Austria; 3 Comprehensive Center for Clinical Neurosciences and Mental Health, Medical University of Vienna, Vienna, Austria; 4 Multiple Sclerosis Center and Neurologia I U, Department of Neuroscience and Mental Health, Azienda Ospedaliero - Universitaria (AOU) Città della Salute e della Scienza di Torino, Torino, Italy; 5 Section of Neurology, Department of Medicine and Surgery, University of Perugia, Perugia, Italy; 6 Department of Neurology, University Medical Center Ljubljana, Ljubljana, Slovenia; 7 Faculty of Medicine, University of Ljubljana, Ljubljana, Slovenia; 8 FH Campus Wien, University of Applied Sciences, Vienna, Austria; 9 Sygehus Sønderjylland, Department of Regional Health Research, University Hospital of Southern Denmark, Hadersleben, Denmark; 10 Department of Neurology, Klinikum Ottakring, Vienna, Austria; 11 Department of Clinical Neuroscience, Institute of Neuroscience and Physiology at Sahlgrenska Academy, University of Gothenburg, Gothenburg, Sweden; 12 Department of Neurology, Aarhus University Hospital, Aarhus, Denmark; 13 Neurology Unit, Department of Translational Medicine, Maggiore della Carità University Hospital, Novara, Italy; 14 Department of Statistics, Faculty of Economics and Statistics, University of Innsbruck, Innsbruck, Austria

**Keywords:** cerebrospinal fluid, kappa free light chain, primary progressive, multiple sclerosis, prognostic biomarker

## Abstract

**Background:**

The kappa free light chain (κ-FLC) index is a well-established biomarker in multiple sclerosis (MS). While the prognostic value of the κ-FLC index has been demonstrated in early relapsing–remitting MS, its prognostic value in primary progressive MS (PPMS) has not yet been investigated.

**Methods:**

In this multicenter, retrospective cohort study, patients diagnosed with PPMS with diagnostic lumbar puncture and clinical follow-up of at least 12 months were recruited from nine MS centers across five countries. At baseline, age, sex, disease duration, and the number of T2 hyperintense (T2L) and contrast-enhancing T1 lesions (CEL) on MRI were determined. κ-FLC was measured using nephelometry/turbidimetry, and the κ-FLC index was calculated as (CSF κ-FLC/serum κ-FLC)/albumin quotient. At follow-up, the occurrence of disability progression and the administration of disease-modifying treatment (DMT) were registered. The primary endpoint was time to disability progression.

**Results:**

A total of 121 PPMS patients were included with a median age of 53 years (25th–75th percentile: 46–59) and a balanced sex distribution (48.8% female). Multivariable Cox regression analysis revealed no significant association between the κ-FLC index and disability progression [hazard ratio (HR) 1.0, p = 0.950]. Prior use of DMT (HR 0.60, p = 0.023) and brain T2L > 9 at baseline (HR 2.22, p = 0.026) were significantly associated with disability progression. The remaining covariates, including age, sex, disease duration, and CEL, showed no significant associations.

**Conclusion:**

The κ-FLC index does not predict disability progression in PPMS, contrasting its growing role as a prognostic biomarker in relapsing MS. This highlights phenotypic differences in MS pathophysiology and underscores the need for prognostic biomarkers in PPMS.

## Introduction

The kappa free light chain (κ-FLC) index is a well-established biomarker for intrathecal immunoglobulin synthesis, included in the 2024 revision of the diagnostic criteria for multiple sclerosis (MS) (1–3). κ-FLC are produced by B cells in excess of intact immunoglobulins and accumulate in the intrathecal compartment in case of inflammatory disorders of the central nervous system (4). It is well-established that the κ-FLC index offers comparable diagnostic sensitivity and specificity as oligoclonal bands (OCB) for the diagnosis of both relapsing–remitting MS (RRMS) and primary progressive MS (PPMS) (5–7). Furthermore, the determination of κ-FLC offers significant advantages over OCB detection, as it can be easily measured using nephelometry/turbidimetry with high reliability (8–10).

In RRMS, several studies have demonstrated the prognostic value of the κ-FLC index. Higher values at disease onset were associated with shorter time to relapse, new MRI activity, disability progression, or cognitive disturbance (11–16). Whether the κ-FLC index also has prognostic value in patients with PPMS has not been investigated so far, which is why we performed the present study.

## Methods

Patients with PPMS from a previous study (6) who had a diagnostic lumbar puncture (LP) and results of cerebrospinal fluid (CSF) analysis, including κ-FLC index, were eligible for inclusion. Additional patients meeting these inclusion criteria were identified by participating centers (**Supplementary Figure S1**). The diagnosis of PPMS was made based on the diagnostic criteria applicable at the time of LP. Furthermore, the 2017 revised McDonald criteria were applied to the whole cohort (17). None of the patients had a history of relapses.

At baseline, age, sex, disease duration, Expanded Disability Status Scale (EDSS), the number of T2 hyperintense (T2L) and contrast-enhancing T1 lesions (CEL) on cerebral MRI, the number of T2L on spinal MRI, and CSF-restricted OCB were determined. During follow-up, disability was assessed using EDSS scores, and disease-modifying treatment (DMT) was recorded at routine clinical visits, in accordance with each center’s routine practice. Due to the heterogeneity in data collection between centers, the EDSS scores were retrieved every 2 years (± 12 months) during follow-up.

### Cerebrospinal fluid analysis

All CSF samples were collected via LP, and serum samples concomitantly within 30 min via venipuncture. All samples were centrifuged at 2,000 *g* for 10 min at room temperature before storage at either −20°C or −80°C (18). CSF analysis was performed for routine diagnostic purposes, including OCB detection by each center using isoelectric focusing followed by IgG immunoblotting/IgG fixation. A detailed description of the methods is provided in **Supplementary Table S1**.

### Determination of κ-FLC index

κ-FLC concentrations in CSF and serum were measured at each center via either nephelometry or turbidimetry using the N Latex kappa FLC (Siemens, Erlangen, Germany) or the Freelite MX™ Kappa Kit (The Binding Site Group Ltd., Birmingham, UK) (19–21).

According to the manufacturer, the lower limit of detection in CSF was 0.034 mg/L for the N Latex kappa FLC Kit. Inter- and intra-assay coefficients of variation (CoV) were <3.3% and <5.5%, respectively. The lot-to-lot variation was ≤4.8%. Linearity was <14.7%. Further details can be found in the work of Velthuis et al. and Pretorius et al. (19, 22). For the Freelite MX™ Kappa Kit, the lower limit of detection was 0.33 mg/L. Inter- and intra-assay CoVs were <7.3% and <4.6%, respectively, according to the manufacturer. The lot-to-lot variation was up to ~20% (23, 24). Further details can be found in the work of Bradwell et al. and White-Al Habeeb et al. (21, 25). The overview of the used methods per center is shown in **Supplementary Table S1** and previous publications (6, 26–33).

Intrathecal κ-FLC synthesis was calculated using the following formula.


$$\kappa -FLC\;index\;=\;\frac{\kappa -FLC_{CSF}\;/\;\kappa -FLC_{Serum}}{Albumin_{CSF}\;/\;Albumin_{Serum}}$$

A κ-FLC index >6.1 was considered “positive”, and a κ-FLC index ≤6.1 was considered “negative” (5).

### Magnetic resonance imaging

Brain and spinal MRI scans were obtained as part of the routine diagnostic work-up. The number of T2L (>/≤ 9) and CEL (≥1/0) of brain MRI, as well as the number of T2L of spinal MRI (≥2/<2), were retrieved from the respective databases of the specialized MS centers. MRI scans were performed on 1.5- or 3-Tesla MRI scanners and rated by experienced local (neuro)radiologists. MRI protocols included contrast-enhanced T1 sequences as well as T2 sequences.

### Definition of disability progression

Disability progression was defined as an EDSS score increase of ≥1.5 for an EDSS baseline score of 0, ≥1.0 for EDSS baseline scores ≥1.0 and ≤5.5, or ≥0.5 for EDSS baseline scores of >5.5 (34), confirmed after 6 months.

### Statistical analysis

Categorical variables were expressed as frequencies and percentages, and continuous variables were displayed as median and 25th–75th percentile, as appropriate. Univariate comparisons were performed using chi-square, Fisher’s test, and the Mann–Whitney U test.

Multivariable Cox regression was employed using time to disability progression as the dependent variable and age (continuous), sex (binary), disease duration (continuous), brain T2L (binary, >/≤ 9), brain CEL (binary, ≥1/0), DMT (binary), and κ-FLC index (continuous) as independent variables.

To visualize the effect of the κ-FLC index, we computed the estimated Cox regression survival probabilities separately for high (>100) and low (≤100) κ-FLC index values (12). We used the median of these high and low κ-FLC index values to plug into the Cox regression to compute the graph.

An *a priori* power analysis for the Cox regression with a significance level of 5%, a power of 80%, and a hazard ratio of two (12) revealed a necessary sample size of 100 patients. We considered a proportion of patients with disability progression of 0.6, and we considered a shorter observation time for patients with disability progression (ratio of observation time in patients with and without disability progression of 0.7).

A p-value <0.05 was considered statistically significant. In Cox regression analysis, according to one-sided hypotheses, that is, increased risk for disability progression, e.g., by higher MRI activity (T2L and CEL) (35) and a lowered risk by DMT (34), one-sided hypothesis testing was used. Thus, a one-sided 95% confidence interval (CI), that is, the lower limit (LL) or upper limit (UL), was shown as appropriate.

All statistical analyses were performed in R (36).

### Ethics statement

The studies involving humans were approved by local ethics committees of participating centers. The studies were conducted in accordance with the local legislation and institutional requirements. The human samples used in this study were acquired as part of previous studies for which ethical approval was obtained (**Supplementary Table S2**). Written informed consent for participation was not required from the participants or the participants’ legal guardians/next of kin in accordance with the national legislation and institutional requirements.

## Results

A total of 121 PPMS patients with a median age of 53 years (46–59) and a balanced sex ratio (48.8% female) were included in the study. All patients fulfilled the 2017 revised McDonald criteria. The patients had a disease duration of 3 (1–6) years and an EDSS score at baseline of 4 (3–5). Brain MRI showed high lesion load (>9 T2L) in 83.6% of patients, and CEL were present in 15.5%. OCB were positive in 108 (89.3%) patients. The median κ-FLC index was 40.0 (14.6–87.8) and considered positive (>6.1) in 112 (92.6%) patients. None of the patients was on DMT at the time of LP; however, DMT was started in 53 (43.8%) patients thereafter. Median follow-up was 5 (3–8) years; i.e., 89.3% of patients had follow-up of at least 2 years.

The details on demographics, clinical characteristics, MRI, and CSF findings are displayed in **Table 1** and **Supplementary Tables S3** and **S4**.

**Table 1:** Demographics, clinical characteristics, MRI, and CSF findings.

Number of patients	121
Age (years)	53 (46–59)
Sex (female)	59 (48.8)
Clinical and MRI characteristics	
Diagnosis made at the time of LP according to McDonald criteria	
2005	6 (5.0)
2010	17 (14.0)
2017	98 (81.0)
Fulfillment of the 2017 revised McDonald criteria^§^	121 (100)
Disease duration at baseline (years)	3 (1–6)
Baseline EDSS	4 (3–5)
Brain T2L (>9) at baseline^#^	97 (83.6)
Brain CEL (≥1) at baseline^#^	17 (15.5)
Spinal T2L (≥2) at baseline^#^	100 (84.0)
Disability progression	79 (65.3)
DMT started during follow-up	53 (43.8)
Time to progression or end of follow-up (years)	3 (2–4)
Cerebrospinal fluid findings	
Q_alb_ (×10^−3^)	5.5 (4.6–7.1)
IgG index	0.7 (0.6–1.0)
OCB (positive)	108 (89.3)
Serum κ-FLC (mg/L)^&^	14.1 (10.5–17.4)
CSF κ-FLC (mg/L)*	3.5 (1.4–6.9)
κ-FLC index	40.0 (14.6–87.8)
κ-FLC index >6.1	112 (92.6)

Data are given as median (25th–75th percentile) and n (%), as appropriate.

CEL, contrast-enhancing lesions; CSF, cerebrospinal fluid; DMT, disease-modifying therapy; EDSS, Expanded Disability Status Scale; LP, lumbar puncture; OCB, oligoclonal band; Q_alb_, CSF/serum albumin ratio; T2L, T2-weighted MRI lesion; κ-FLC, kappa free light chain.

^§^ 2017 revised McDonald criteria were fulfilled by at least 1 year of disability progression in all patients and i) ≥2 T2L in the spinal cord, ≥1 T2L in one or more of the typical brain regions [periventricular, (juxta-)cortical, or infratentorial], positive OCB (n = 86); or ii) ≥1 T2L in one or more of the typical brain regions, positive OCB (n = 21); or iii) ≥2 T2L in the spinal cord, positive OCB (n = 1); or iv) ≥2 T2L in the spinal cord, ≥1 T2L in one or more of the typical brain regions (n = 13).

^#^ Brain MRI was available in 116 and contrast-enhanced brain MRI in 110 patients. Spinal MRI was available in 119 patients.

* All κ-FLC concentrations were above the lower detection limit.

^&^ One patient had high serum κ-FLC concentration of 81.1 mg/L. In this patient, the κ-FLC index was negative (1.39), and OCB was negative.

### Progressive vs. stable patients

A total of 79 (65.3%) patients had disability progression during follow-up. In univariate analyses, age, sex, disease duration, EDSS, T2L, and CEL at baseline were similar between progressive and stable PPMS patients. The proportion of patients on DMT during follow-up was comparable between the two groups (**Table 2**). The median κ-FLC index determined at baseline was numerically higher in progressive patients (43.7; 15.5–108.2) compared to the stable group (26.1; 13.9–75.8); however, this difference did not reach statistical significance (p = 0.328; **Table 2**).

**Table 2:** Demographics, clinical characteristics, MRI, and CSF findings in progressive and stable PPMS patients.

	Progressive	Stable	p-Value
Number of patients	79	42	
Age (years)	53 (45–59)	54 (48–60)	0.417^1^
Sex (female)	39 (49.4)	20 (47.6)	1.000^2^
Clinical and MRI characteristics			
Disease duration at baseline (years)	3 (1–6)	3 (2–6)	0.678^1^
Baseline EDSS	4 (2.5–5)	3.5 (3.0–4.5)	0.795^1^
Brain T2L (>9) at baseline^#^	67 (87.0)	30 (76.9)	0.262^2^
Brain CEL (≥1) at baseline^#^	13 (16.9)	4 (10.3)	0.550^2^
Spinal T2L (≥2) at baseline^#^	68 (88.3)	32 (76.2)	0.087
DMT started during follow-up	31 (39.2)	22 (52.4)	0.232^2^
Cerebrospinal fluid findings			
OCB (positive)	71 (89.9)	37 (88.1)	0.765^2^
Serum κ-FLC (mg/L)	14.1 (10.1–17.2)	14.1 (11.6–17.6)	0.804^1^
CSF κ-FLC (mg/L)	3.6 (1.4–7.3)	3.1 (1.3–6.2)	0.334^1^
κ-FLC index	43.7 (15.5–108.2)	26.1 (13.9–75.8)	0.328^1^
κ-FLC index >6.1	75 (94.9)	37 (88.1)	0.273^2^
Follow-up			
Time to progression or end of follow-up (years)	2 (2–4)	3 (2–6)	0.002

Data are given as median (25th–75th percentile) and n (%), as appropriate.

EDSS, Expanded Disability Status Scale; T2L, T2-weighted MRI lesion; CEL, contrast-enhancing lesion; DMT, disease-modifying therapy; Q_alb_, CSF/serum albumin ratio; OCB, oligoclonal band; κ-FLC, kappa free light chain; CSF, cerebrospinal fluid.

Group comparisons were performed using ^1^ Mann–Whitney U test or ^2^ chi-square/Fisher’s test.

^#^ Brain MRI scans available for 77 progressive and 39 stable patients, and contrast-enhanced MRI available for 74 progressive and 36 stable patients. Spinal MRI scans available for 77 progressive and 42 stable patients.

### κ-FLC index does not predict disability progression in PPMS

**Figure 1:**
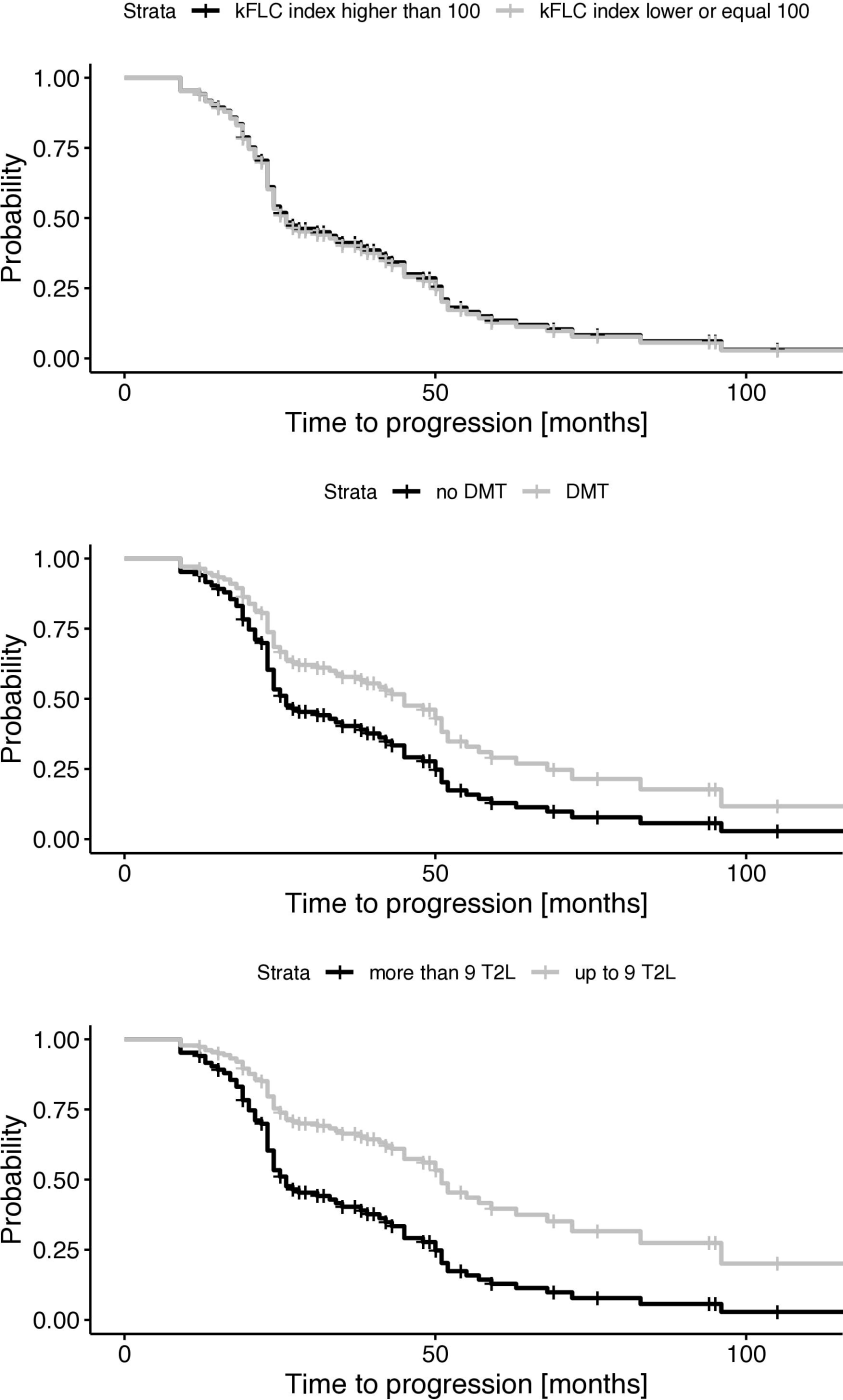
Probability of disability progression dependent on κ-FLC index, DMT, and MRI lesion load. FLC, free light chain; DMT, disease-modifying therapy; T2L, hyperintense lesion on T2-weighted MRI; κ-FLC, kappa free light chain.

Multivariable Cox regression analysis showed that the κ-FLC index was not a statistically significant predictor of time to disability progression [hazard ratio (HR) 1.0, p = 0.950]. In contrast, DMT use (HR 0.60, UL-CI: 0.91, p = 0.023) and brain MRI T2L at baseline (HR 2.22, LL-CI: 1.13, p = 0.026) were predictors of disability progression. The remaining variables, age, sex, disease duration, and CEL showed no statistically significant association (**Figure 1**, **Table 3**). Further analyses, also including OCB status (**Supplementary Table S5**) and T2L on spinal MRI (**Supplementary Table S6**), yielded qualitatively the same results.

**Table 3:** Cox regression analysis identifying predictors of disability progression.

Variable	Estimate	Standard error	HR	p-Value^2^	95% CI^2^	
					Lower limit	Upper limit
κ-FLC index (per increase of 10)	−0.002	0.020	0.998	0.950	–	
Age (years)	0.002	0.012	1.003	0.417	–	
Sex (ref: male)	−0.071	0.243	0.932	0.385	–	
Baseline brain MRI T2 lesion load (ref: ≤9)	0.796	0.411	2.217	**0.026**	1.127	
Baseline brain CEL (ref:<1)	0.276	0.349	1.318	0.215	–	
DMT^1^ (ref: no treatment)	−0.506	0.253	0.603	**0.023**	–	0.914
Disease duration (years)	−0.008	0.036	0.992	0.412	–	

R^2^ = 0.103.

FLC, free light chain; CEL, contrast-enhancing lesion; CI, confidence interval; DMT, disease-modifying therapy; HR, hazard ratio; MRI, magnetic resonance imaging; κ-FLC, kappa free light chain.

^1^DMT administration until disability progression or until end of observation in stable patients.

^2^One-sided p-value of<0.05 was considered statistically significant; therefore, one-sided 95% CI is shown.

P-values <0.05 were marked bold.

## Discussion

Here, we investigated whether the κ-FLC index, a quantitative biomarker of intrathecal inflammation, predicts disability progression in patients with PPMS. Our study, including a total of 121 patients, revealed no significant association, even after adjusting for established covariates.

The prognostic value of intrathecal inflammation has been shown by a multitude of studies in relapsing–remitting MS through both the κ-FLC index and OCB (4). In PPMS, the prognostic value of the κ-FLC index has not yet been investigated, and previous studies using OCB have not found any association with the disease course (37, 38).

A possible explanation why intrathecal inflammation, as determined by OCB, was not prognostic in PPMS, but is a clear predictor in RRMS, could be that the inflammatory extent and its contribution to disease evolution are lower in PPMS compared to RRMS (39). We had hypothesized that the κ-FLC index, in contrast to OCB, could exert some prognostic capabilities in PPMS due to the previously reported superiority of the κ-FLC index over OCB in terms of the prognosis of MS disease course (12). While OCB were detected in 95% of patients with relapses during follow-up, OCB were also positive in 86% of non-relapsing patients. The κ-FLC index, as a continuous variable, overcame the weak performance of OCB by further stratification. In the subgroup of OCB-positive patients only, the κ-FLC index was still statistically significantly higher in patients with relapses compared to those without relapses, and testing for log-likelihood reduction by including either the κ-FLC index or OCB in the prognostic model clearly confirmed the superior prognostic value of the κ-FLC index (12). Furthermore, OCB only detect intrathecal IgG production (40), while the κ-FLC index captures intrathecal synthesis from IgG, IgA, and IgM (41, 42). It could have been that this broader spectrum enhanced sensitivity to intrathecal immune responses, as a prognostic value of IgM OCB in PPMS has been reported (37).

Ultimately, we did not observe a statistically significant prognostic value of the κ-FLC index in patients with PPMS (**Figure 1A**). *A priori*, we performed a power analysis for the Cox regression (as specified in detail in the methods), considering a power of 80% and a hazard ratio of two. The effect size of the κ-FLC index in RRMS was usually high (12), and for other variables, i.e., DMT and MRI T2L, we did observe a difference between progressing and non-progressing patients in the present analysis (**Figures 1B, C**) (34, 35). Of course, we cannot exclude that having more patients would have uncovered minor prognostic effects of the κ-FLC index.

Interestingly, the κ-FLC index was higher in patients with CEL (43.7) compared to patients without CEL (31.5) by univariate analysis (p = 0.049). An interaction effect in the Cox regression model between CEL and κ-FLC index was considered in order to investigate whether the κ-FLC index provides additional prognostic value; e.g., only in patients with contrast enhancement it did not show any effect. However, for this analysis, the number of patients (with CEL, n = 17) was too small. Future studies could explore κ-FLC’s utility in PPMS patients with concomitant inflammatory activity. The stratification of patients, those with CEL and high κ-FLC index, may gain further utility, for example, for the evaluation of treatment response, as it is known that patients with inflammatory activity benefit more from B-cell depletion therapies (34).

Our study has several limitations that warrant consideration. It was a retrospective study with all inherent attributes, e.g., the inclusion of patients depended on the availability of samples and follow-up data. Furthermore, time intervals between consecutive clinical visits and MRI protocols used for the determination of baseline T2L and the presence of CEL differed between centers, too. Differences in sampling handling (e.g., processing of fresh versus thawed samples) and laboratory methods used for κ-FLC detection (nephelometry vs. turbidimetry, or polyclonal vs. monoclonal detection antibody) may have led to variability in absolute κ-FLC values, although the calculation of the κ-FLC index does minimize this effect (43). While after frozen sample storage, some decreases in absolute κ-FLC concentrations in CSF and serum have been observed, these changes are evened out using CSF/serum ratios of κ-FLC (and albumin) when calculating the κ-FLC index (43). Also, OCB positivity may vary due to different detection methods (e.g., IgG immunoblotting vs. silver staining). Notably, none of the patients had received DMT at the time of LP, thereby eliminating potential confounding effects of immunomodulatory treatment on κ-FLC index levels (44–46). We also would like to state that disability progression was based solely on the EDSS. The assessments of upper extremity function or cognition were not available. Including further modalities that increase sensitivity for the detection of disability progression may influence the assessment of the prognostic value of the κ-FLC index. Furthermore, we could not consider longitudinal changes of MRI lesions for our analysis, as follow-up MRIs were not regularly performed. Investigation of the prognostic value of the κ-FLC index using a more sensitive endpoint, such as MRI activity, should be addressed by further research. This would also allow us to consider progression independent of MRI activity.

Although the κ-FLC index did not demonstrate a significant prognostic value in PPMS, this study provides a relevant piece of evidence for the interpretation of the κ-FLC index, a biomarker that is already used in clinical routine.

## Data Availability

The raw data supporting the conclusions of this article will be made available by the authors, without undue reservation.
