# Supplementary material for: Prognostic value of kappa free light chain index in patients with primary progressive multiple sclerosis
Source: Front Immunol. 2025 Nov 7;16:1658182. doi: 10.3389/fimmu.2025.1658182 (PMC12634365; doi:10.3389/fimmu.2025.1658182)
Supplement: Supplementary file 1 [file DataSheet1.pdf]

Figure S1: Inclusion of patients

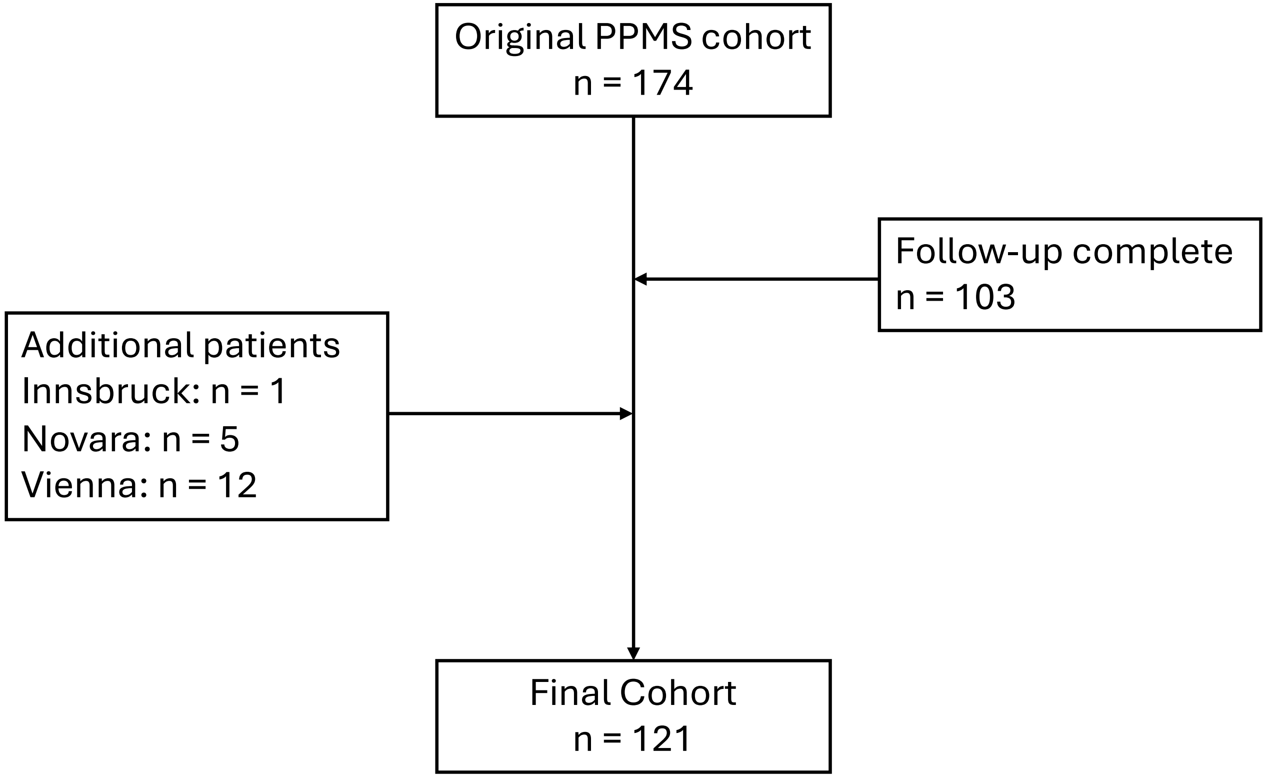

Legend:
Abbreviations: PPMS, primary progressive multiple sclerosis

Table S1: Laboratory methods for OCB detection and κ-FLC measurement by center

**Center**	**OCB detection**			**κ-FLC measurement**	
	**Method**	**Technique**	**Detection**	**Platform**	**κ-FLC assay**
**Turin**	Sebia	IEF (agarose gel)	IgG immunofixation	Siemens BNII	N Latex
**Novara**	Sebia	IEF (agarose gel)	IgG immunoblotting	Siemens BNII	N Latex
**Perugia**	Sebia	IEF (agarose gel)	IgG immunofixation	Siemens BNII	Freelite
**Aarhus**	Sebia	IEF (agarose gel)	IgG immunofixation	SPAPLUS	Freelite
**Ljubljana**	In-house assay	IEF (agarose gel)	IgG immunoblotting	Siemens BN Prospec	N Latex
**Gothenburg**	In-house assay	IEF (polyacrylamide gel)	Silver staining	Atellica	N Latex
**Vienna A***	In-house assay	IEF (agarose gel)	IgG immunoblotting	Atellica	N Latex
**Vienna B#**	Helena SPIFE	IEF (agarose gel)	IgG immunoblotting	Behring Prospec	Freelite
**Innsbruck**	In-house assay	IEF (polyacrylamide gel)	IgG immunoblotting	Atellica	N Latex

Legend:
Methods used in patients * published in Presslauer et al. 2008 and 2016, and # additionally recruited later. *Abbreviations*: IEF, isoelectric focusing

Table S2: Cohorts per center with corresponding ethics committees' approvals

Center	Publication	Year	Ethics information
**Turin**	Cavalla et. al.	2020	Local ethics committee - Comitato Etico interaziendale AOU Città della Salute e della Scienza di Torino, AO Ordine Mauriziano, ASL Città di Torino (approval number 003842021).
**Novara***	Vecchio et.al	2020	Local Ethics Committee - Comitato Etico Interaziendale AOU "Maggiore della Carità" di Novara, ASL BI, ASL NO, ASL VCO (approval number CE 190/19)
**Perugia**	Gaetani et.al	2020	Local Ethics Committee (approval number 2320/14)
**Aarhus**	Christiansen et.al.	2018	Central Denmark Region Committees on Health Research Ethics (approval number 20090210)
**Ljubljana**	Emersic et.al	2019	National Medical Ethics Committee of Slovenia (approval number 0120-46212015-2 and 0120-308/2021/3)
**Gothenburg**	Rosenstein et.al	2021	Swedish Ethical Review Agency (approval number 2020-06851)
**Vienna***	Presslauer et.al	2008/2016	Vienna Ethics Committee (approval number 1368/2023)
**Innsbruck***	Hegen et. al.	2023	Innsbruck Ethics Committee (approval number 1269/2022)

Legend:
* In addition, patients were included who had not been previously published.

Table S3: Immunotherapies started during follow-up

Immunotherapy	Number of patients
Anti-CD20 monoclonal antibody treatment	
**Ocrelizumab**	27
**Rituximab**	15
Other treatments	
**Interferon-beta**	3
**Azathioprine**	3
**Siponimod**	2
**Glatiramer acetate**	1
**Teriflunomide**	1
**AHSCT**	1

Legend:
Abbreviations: AHSCT, autologous hematopoietic stem cell transplant; MS, multiple sclerosis

Table S4: OCB status according to different methods

	OCB positive	OCB negative
**In house and in-vitro-diagnostics**		
**Sebia**	40 (87.0)	6 (13.0)
**In house**	62 (89.9)	7 (10.1)
**Helena**	6 (100)	0 (0)
**IgG immunoblotting/ -fixation and silver staining**		
**IgG Immunoblotting**	57 (89.0)	7 (11.0)
**IgG Immunofixation**	34 (87.2)	5 (12.8)
**Silver staining**	17 (94.4)	1 (5.6)
**Different centers**		
**Turin**	27 (87.1)	4 (12.9)
**Novara**	6 (85.7)	1 (14.3)
**Perugia**	3 (75.0)	1 (25.0)
**Aarhus**	4 (100)	0 (0)
**Ljubljana**	23 (88.5)	3 (11.5)
**Gothenburg**	17 (94.4)	1 (5.6)
**Vienna A**	9 (81.8)	2 (18.2)
**Innsbruck**	13 (92.9)	1 (7.1)
**Vienna B**	6 (100)	0 (0)
**Total**	108 (89.3)	13 (10.7)

Legend:
n (%). *Abbreviations:* OCB, oligoclonal bands

Table S5: Cox regression analysis identifying predictors of disability progression including OCB

Variable	Estimate	Standard error	HR	P-value[2]	95%-CI[2]	
					Lower Limit	Upper Limit
**κ-FLC index** (per increase of 10)	-0.003	0.015	0.999	0.924	-	
**Age** (years)	0.003	0.012	1.003	0.391	-	
**Sex** (ref: male)	-0.084	0.244	0.919	0.365	-	
**Baseline brain MRI T2 lesion load** (ref: ≤9)	0.778	0.414	2.177	**0.030**	1.103	
**Baseline brain CEL** (ref: <1)	0.262	0.349	1.300	0.226	-	
**DMT**[1] (ref: no treatment)	-0.491	0.256	0.612	**0.027**	-	0.931
**Disease duration** (years)	-0.006	0.036	0.994	0.437	-	
**OCB** (ref: negative)	0.186	0.403	1.205	0.322		

R^2= 0.106

Legend:

[1] DMT administration until disability progression, or until end of observation in stable patients.

[2] One-sided p value of <0.05 was considered as statistically significant; therefore, 1-sided 95% CI is shown.

Abbreviations: FLC, free light chain; CEL, contrast enhancing lesion; CI, confidence interval; DMT, diseases modifying therapy; MRI, magnetic resonance imaging; OCB, oligoclonal bands; HR, hazard ratio

Table S6: Cox regression analysis identifying predictors of disability progression including spinal T2 MRI lesions

Variable	Estimate	Standard error	HR	P-value[2]	95%-CI[2]	
					Lower Limit	Upper Limit
**κ-FLC index** (per increase of 10)	-0.002	0.017	0.999	0.954	-	-
**Age** (years)	-0.001	0.012	0.999	0.955	-	-
**Sex** (ref: male)	-0.036	0.251	0.965	0.443	-	-
**Baseline brain MRI T2 lesion load** (ref: ≤9)	0.794	0.416	2.211	**0.028**	1.115	-
**Baseline brain CEL** (ref: <1)	0.284	0.360	1.329	0.215	-	
**DMT[1]** (ref: no treatment)	-0.617	0.263	0.539	**0.009**	-	0.831
**Disease duration** (years)	-0.008	0.038	0.992	0.915	-	-
**Baseline spinal MRI lesion load** (ref: <2)	0.589	0.371	1.802	0.056	-	-

R^2= 0.148

Legend:
[1] DMT administration until disability progression, or until end of observation in stable patients.
[2] One-sided p value of <0.05 was considered as statistically significant; therefore, 1-sided 95% CI is shown.
Abbreviations: FLC, free light chain; CEL, contrast enhancing lesion; CI, confidence interval; DMT, diseases modifying therapy; HR, hazard ratio; MRI, magnetic resonance imaging
